# Scrub Typhus in the Torres Strait Islands of North Queensland, Australia

**DOI:** 10.3201/eid0904.020509

**Published:** 2003-04

**Authors:** Antony G. Faa, William J.H. McBride, Gaynor Garstone, Robert E. Thompson, Peter Holt

**Affiliations:** * Thursday Island Hospital, Queensland, Australia; † Cairns Base Hospital, Queensland, Australia; ‡ Darnley Island Health Centre, Queensland, Australia

**Keywords:** *Orientia tsutsugamushi*, scrub typhus, Australia, Queensland, Papua New Guinea, climate, azithromycin

## Abstract

Scrub typhus, caused by *Orientia tsutsugamushi*, occurs throughout Southeast Asia. We descript ten cases that occurred in the Torres Strait islands of northern Australia during 2000 and 2001. Preceding heavy rain may have contributed to the outbreak. The successful use of azithromycin in two pediatric patients is also reported.

Scrub typhus is a rickettsial disease caused by the bacteria *Orientia tsutsugamushi* and is transmitted to humans from the bite of a larval trombiculid mite. The disease is distributed throughout Southeast Asia and is restricted by the distribution of the host mite (*1*). Scrub typhus has been recognized as being endemic to north Queensland since the 1920s (*2*). More recently, the disease has been recognized in the Northern Territory (*3*) and the northwest of western Australia (*4*). In north Queensland the disease is most commonly recognized between Ingham and Cooktown. In a recent review of cases from north Queensland, none were reported outside of this area (*5*). In March 2000, scrub typhus was diagnosed in a visitor to Torres Strait from Papua New Guinea. Additional cases were subsequently diagnosed in residents of Torres Strait. We describe a cluster of cases from Darnley Island in the Torres Strait. This island, and possibly others in the Torres Strait, should now be considered as scrub typhus–endemic foci.

## Case Studies

A 35-year-old man, a visitor from Papua New Guinea, arrived at the Darnley Island Health Centre in March 2000, complaining of headache and symptoms of fever. He had arrived on Darnley Island 24 days before onset of illness. He began a course of oral quinine for suspected malaria but remained ill and was transferred to Thursday Island Hospital. He was noted to be febrile (40.2°C) and had marked conjunctival injection. No obvious focus of infection was evident. The day after admission he was still febrile and was given Timentin (ticarcillin plus clavulinic acid), gentamicin, and doxycycline. His condition gradually improved, and the fever subsided in 5 days. Scrub typhus serologic test results were strongly positive (Table, patient 1).

**Table:** Demographic, clinical and serologic data for patients with scrub typhus, Torres Strait islands, Queensland, Australia

Patient no.	Age (y)/sex	Date	Island	Eschar	*Orientia tsutsugamushi* antibody titer^a^ (d after onset)				
					0–7	8–14	15–21	22–28	>29
1	35/male	Mar 2000	Darnley	No		8,192			8,192
2	19/male	May 2000	Murray	Yes	<64	4,096			4,096
3	29/male^b^	Aug 2000	—	No	1,024	1,024			1,024
4	28/female	Jan 2001	Darnley	Yes		NR		512	
5	9/male	Jan 2001	Darnley	No			256		256
6	14/female	Jan 2001	Darnley	No		512	512		
7	36/male	Jan 2001	Darnley	Yes	NR		512		
8	19/male	Jan 2001	Darnley	No	64^c^			256	
9	5/male	Feb 2001	Darnley	Yes		>1,024	>1,024		
10	30/male^b^	Apr 2001	—	Yes	NR^c^	128			

^a^Serologic tests were performed by using an immunofluorescent antibody method; NR, nonreactive.

^b^Patient visited several islands in 3 weeks before becoming ill.

^c^Polymerase chain reaction for scrub typhus positive (by using primers for 56-kDa antigen gene).

A 19-year-old patient (patient 2) was transferred from Murray Island to the Thursday Island Hospital in May 2000. He had been ill for 12 days with fever, frontal headache, and intermittent vomiting. He had a documented temperature of 40.1°C before admission to hospital and had been treated symptomatically with aspirin and metoclopromide. He had not left Murray Island for several months and had never traveled to Papua New Guinea. He denied knowledge of any mite bites. On admission he looked ill and complained of lethargy; his temperature was 39.4°C. He had slight conjunctival injection and mild tenderness in the right upper quadrant of his abdomen. A typical black eschar approximately 0.5 cm in diameter was found on his upper right thigh with associated tender right inguinal lymphadenopathy. Tests indicated a normal leukocyte count (9.8 x 10^9^/L), thrombocytopenia (platelet count 79,000/µL), increased creatinine (0.14 mmol/L), and abnormal liver function tests (γ-glutamyl transferase 55 U/L, normal <50; alanine aminotransferase 280 U/L, normal <45; aspartate aminotransferase 223 U/L, normal <40). He received doxycyline, became afebrile after 48 hours, and made an uncomplicated recovery

In August 2000, a 29-year-old man (patient 3) arrived at the primary health center on Thursday Island with a 5-day history of fevers, sweats, lethargy, and headache. As a field worker with the local electricity authority, he had visited several of the outer Torres Strait islands (Yorke, Darnley, Mabuaig, Yam, Badu, and Stephen) in the 3 weeks before becoming ill. These visits included a 3-day trip to Darnley Island 2 weeks before his fevers began. Some of his work involved clearing vegetation from the fence lines of the local power plants. On examination he looked ill, but no abnormal physical signs were found. He did not have an eschar or rash. He was seen 5 days later and still had a temperature of 40°C. He was not treated with antibiotics, and his fever settled spontaneously during the 3 weeks.

**Figure 1:**
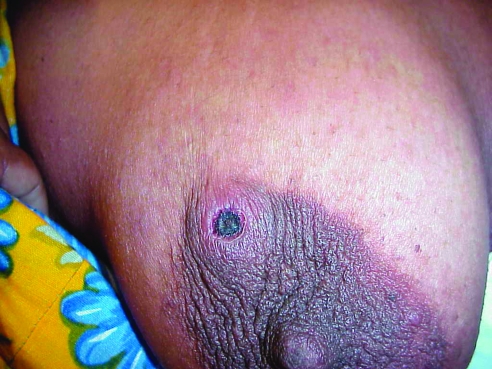
Eschar on the breast of a patient with scrub typhus during an outbreak on Darnley Island, Torres Strait.

A 28-year-old woman (patient 4) arrived at Darnley Island Health Centre in January 2001 with her 9-year-old son (patient 5); both complained of being ill for approximately 1 week. They reported fevers, headache, and generalized myalgia. The child also complained of vomiting and was noted to have a dry cough with some rhinorrhea. He was started on a course of amoxicillin. The next day, when seen at the visiting doctors clinic, both mother and son were given oral quinine to treat possible malaria. Over the next few days, both remained febrile with temperatures up to 40°C. They were transferred to Thursday Island Hospital. The mother had a typical black eschar on her left breast (Figure 1) with associated regional lymphadenopathy. She also had conjunctival injection. Oral doxycycline was administered, and the fever resolved over 3 days. Her son was treated with azithromycin (250 mg a day for 3 days) and became afebrile within 24 hours.

In the next 3 weeks, four more patients (one adult [patient 7], two teenagers [patients 6 and 8], and one 5-year-old child [patient 9]) arrived at Darnley Island Health Centre with nonspecific febrile illnesses associated with headache. Two had eschars (one located on the scrotum and another on the scalp) with associated regional lymphadenopathy. One patient had conjunctival injection, and another had a fine truncal rash. Two were treated with oral doxycycline and became well soon after starting treatment. Patient 6 was successfully treated with azithromycin (1,000 mg as a single dose). Patient 9 had been given a course of oral and intramuscular penicillin before scrub typhus was suspected and remained ill with fevers and abdominal pain (with right upper quadrant tenderness). He was transferred to the hospital 2 weeks after being initially seen but was afebrile on admission and remained well with no additional treatment. An additional case of scrub typhus (patient 10) was documented in April 2001.

### Epidemiologic Findings and Community Interventions

After recognition of this cluster of scrub typhus cases, a doctor and indigenous health worker traveled to Darnley Island to interview patients and educate the local community about the disease. The six patients had not left Darnley Island in the 6 weeks before becoming ill. The patients lived on different parts of the island, and no common area was visited before illness onset. Patient 4 was an exception and had harvested mangoes in an area of jungle close to her house where her son (patient 5) played. The transmission of scrub typhus on Darnley Island probably occurred in the densely vegetated areas close to most domestic dwellings on the island. A public meeting was held, which focused on increasing the general awareness of the signs and symptoms of scrub typhus and the importance of early diagnosis and treatment. The meeting included local indigenous health workers. Aspects of personal protection against mite bites were also stressed, including wearing protective boots and trousers and using insect and mite repellants when going into potential areas of transmission.

## Conclusions

**Figure 2:**
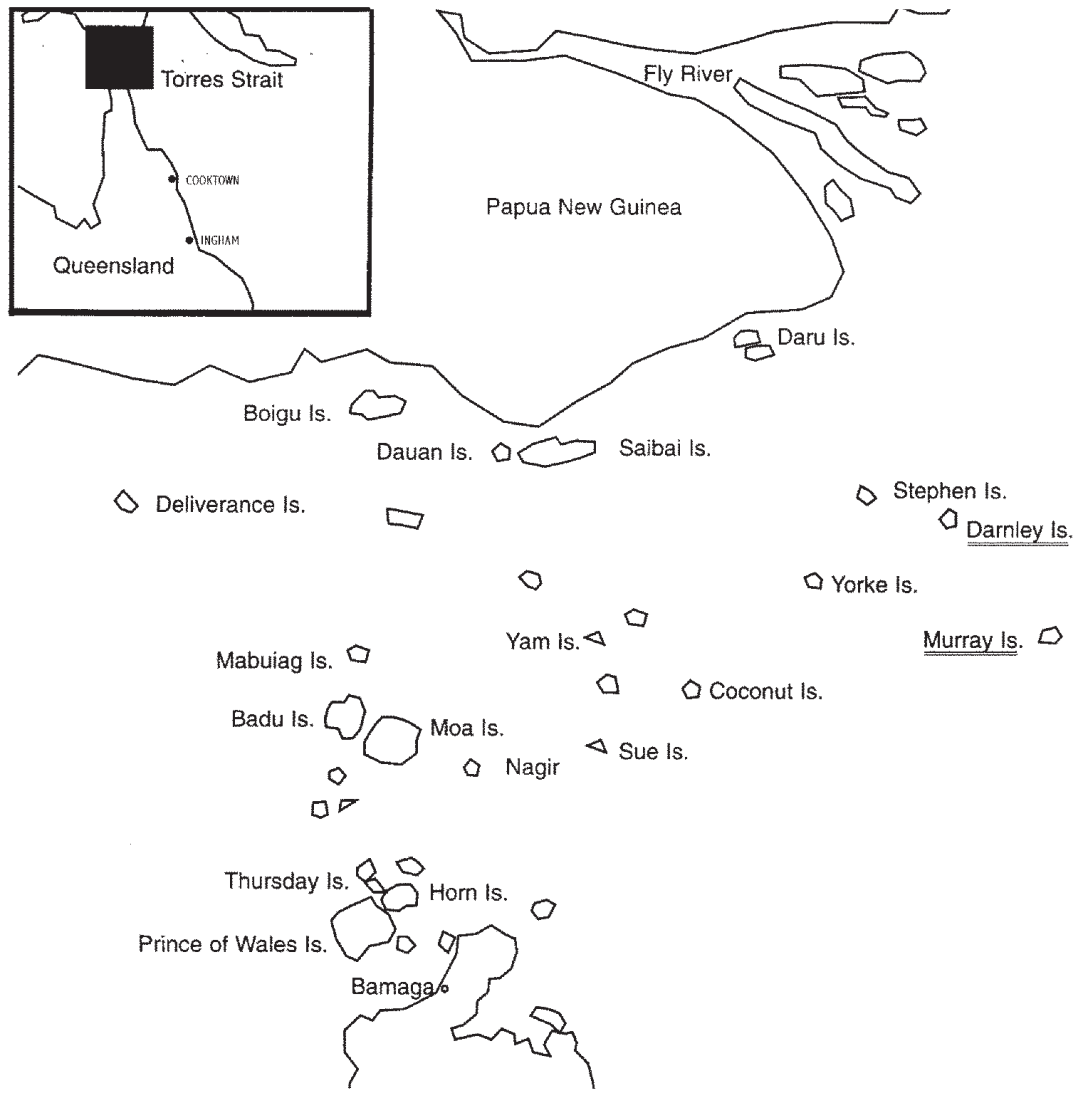
Map of Torres Strait islands showing areas of scrub typhus transmission, Darnley and Murray Islands, Torres Strait, 2000–2001.

The Torres Strait islands (Figure 2) lie between Cape York Peninsula in Queensland and Papua New Guinea. The islands have a population of approximately 9,000 mainly indigenous inhabitants. Approximately half of the people live on or near Thursday Island, which is the main commercial and government center. A 38-bed general hospital is located there. The other half of the population lives on the 15 outer island communities that stretch between the Australian and Papua New Guinea mainland. Health centers are located on each of these islands and are staffed by remote area nurses, indigenous healthcare workers, or both.

The cases of scrub typhus infection we describe are not the first to be described from the Torres Strait islands. Scrub typhus was previously reported in the 1950s, with a single case from Hammond Island (near Thursday Island), and another in a diver from a lugger “near Darnley Island” (*2*).

We describe at least two islands where transmission of scrub typhus occurred in the Torres Strait during the wet seasons of 2000 and 2001. One infection occurred on Murray Island, and seven occurred on Darnley Island. Another patient lived on Thursday Island but traveled to several outer islands in the weeks before becoming ill and probably acquired his infection on Darnley Island. The occurrence of six cases of scrub typhus on Darnley Island over a 3-week period is important, representing the infection of nearly 2% of the total population (375) (*6*). Rainfall for the region was well above average in November and December 2000, the beginning of the wet season (*7*). The resulting dense vegetation would have provided favorable conditions for the transmission of disease. We believe that *O. tsutsugamushi* has probably existed in the region for many decades, rather than having been recently introduced, and climatic conditions favored the transmission to humans in the early part of 2001.

Our series included two children and one young teenager. A 9-year-old boy and 14-year-old girl responded well to treatment with azithromycin. Azithromycin has been used successfully in the treatment of scrub typhus in a small number of pregnant women (*8*), but to our knowledge these are the first published cases of its clinical use in children with the disease.
